# Characteristics, Treatment, Outcomes, and Survival in Neuroendocrine G1 and G2 Pancreatic Tumors: Experiences From a Single Tertiary Referral Center

**DOI:** 10.3389/fendo.2021.657698

**Published:** 2021-04-13

**Authors:** Jan Calissendorff, Freja Bjellerup-Calissendorff, Robert Bränström, C. Christofer Juhlin, Henrik Falhammar

**Affiliations:** ^1^Department of Endocrinology, Karolinska University Hospital, Stockholm, Sweden; ^2^Department of Molecular Medicine and Surgery, Karolinska Institutet, Stockholm, Sweden; ^3^Department of Pathology, Västmanland County Hospital, Västerås, Sweden; ^4^Center for Clinical Research, Uppsala University, Västmanland County Hospital, Västerås, Sweden; ^5^Department of Breast, Endocrine and Sarcoma Surgery, Karolinska University Hospital, Stockholm, Sweden; ^6^Department of Clinical Pathology and Cytology, Karolinska University Hospital, Stockholm, Sweden; ^7^Department of Oncology-Pathology, Karolinska Institutet, Stockholm, Sweden

**Keywords:** neuroendocrine neoplasia, pancreatic, treatment, outcome, survival, Ki-67, size, functionality

## Abstract

**Purpose:**

Neuroendocrine tumors of the pancreas (Pan-NETs) are usually hormonally inactive with a capacity to metastasize. Since Pan-NETs are rare, more knowledge is needed.

**Methods:**

We reviewed all patients’ medical files with Pan-NET treated at a tertiary center (2006-2019). Grade 1 (G1) and grade 2 (G2) tumors were compared. The latter group was subdivided arbitrarily based on proliferation index into G2a (3-9.9%) and G2b (10-19.9%).

**Results:**

We found 137 patients (76 females, 61 males; G1 n=66, G2 n=42), the median age at diagnosis 61 years (interquartile range (IQR) 50–71), and tumor size 2 cm (1.3–5 cm). The initial surgery was performed in 101 patients. The remaining (n=36) were followed conservatively. Metastatic disease was evident in 22 patients (16%) at diagnosis while new lesions developed in 13 out of 22 patients (59%). In patients without previous metastatic disease, progressive disease was discovered in 29% of G1 vs. 55% of G2 patients (*P*=0.009), 47% of G2a vs. 75% of G2b patients (NS). Survival was poorer in patients with metastasis at diagnosis vs. those with local disease (*P*<0.001). During follow-up of 74 months, Pan-NET related death was found in 10 patients. Survival was not different between G1 vs. G2 or G2a vs. G2b, or if tumors were functional. Size ≤2 cm was associated with a better outcome (*P*=0.004). During the follow-up of small tumors (≤2 cm, n=36) two were resected.

**Conclusion:**

In small non-functional Pan-NETs, active surveillance is reasonable. Progressive disease was more common in G2, but survival was similar in G1, G2 and between G2 subgroups. Survival was poorer in patients with metastasis at diagnosis.

## Introduction

The yearly incidence of cases with neuroendocrine pancreatic tumors (Pan-NETs) is 0.5-0.8/100 000 (1). Of these, only a minority is functional, i.e., hormone-secreting, while 60-90% are non-functional (2–4). When non-functional lesions are symptomatic, the most common presenting symptoms are abdominal pain (35–78%), anorexia and nausea (45%), as well as weight loss (20–35%) (5). Symptoms in functional tumors depend on the many variants of hormone-producing Pan-NETs and which hormones are being secreted (6).

Pan-NETs can also be incidental findings when radiology is performed for other reasons, i.e., pancreatic incidentalomas. There is an increase in patients diagnosed with Pan-NETs, mostly in early stages, probably secondary to more frequent imaging (7). Most functioning and non-functioning Pan-NETs occur sporadically, but they can also be diagnosed in the work-up of patients with familial syndromes such as multiple endocrine neoplasia type 1 (MEN1) or von Hippel Lindau disease (vHL). Neuroendocrine lesions in the pancreas are often slowly growing with the potential to metastasize (2). Surgery should generally be performed if tumors are hormonally active and if the tumor is larger than 2 cm. There are controversies whether to operate or not in non-secreting tumors of 1-2 cm in size (8). The best prognostic factor for progression is Ki-67 (9). Neoplasms with neuroendocrine features are graded by proliferation index from G1 (Ki-67 index <3%), G2 (3-20%), and G3 (>20%). G3 tumors can furthermore be classified as either Pan-NET G3 or pancreatic neuroendocrine carcinoma (Pan-NEC). The former category often exhibits well-differentiated histology and mutations in either *DAXX* or *ATRX*, while Pan-NECs display poor differentiation and mutations in *TP53* and/or *RB1* (10).

Pan-NETs are clinically heterogeneous and can exhibit indolent behavior but also progress to more clinically aggressive tumors. The prognostication mainly relies on the Ki-67 proliferation index but also depend on functionality and tumor size. Irrespectively, long-term follow-up of patients with Pan-NETs is required.

This study aimed to describe a sizable institutional series of Pan-NETs from biochemical, surgical, and histopathological features and relate these parameters to patient outcome and survival.

## Methods

This retrospective investigation includes 137 patients with Pan-NETs treated between 2006 and 2019 at the Karolinska University Hospital, Stockholm, Sweden. The catchment area is designated for highly specialized care, including pancreatic surgery of more than 2 million inhabitants (11, 12). All hospital admissions and out-patient visits in Sweden are coded with the International Classification of Diseases version 10 (ICD-10) codes by the attending physician and are stored in both local and national databases (13). All patients with the ICD-10 codes C25.4 (malignant neoplasm of pancreas, islet of Langerhans), C25.9 (malignant neoplasm of pancreas, unspecified), and D13.7 (benign neoplasm of the pancreas) were selected. All the relevant electronic medical files of the patients with Pan-NET were reviewed manually. The date of diagnosis was defined as the time of the multidisciplinary meeting, or the day of surgery if the Pan-NET diagnosis was made first after surgery. We noted radiology, tumor size, biochemical tests, initial and repeated Ki-67 indexes obtained through histopathological investigations in operated patients. The Ki-67 was calculated by counting the percentage of positive tumor nuclei in 2000 cells in hot spot areas. The cohort was divided into G1 cases (tumors with a Ki-67 index of <3%) and G2 group (Ki-67 index between 3–20%). No Pan-NET G3 cases or Pan-NECs were included. As the span in G2 tumors are wide the G2 group was further arbitrarily subdivided into G2a (Ki-67 index 3–9.9%) and G2b (Ki-67 index 10–19.9%). The initial grade could not be evaluated in 36 patients diagnosed by imaging and biochemical testing. These patients were followed clinically with repeated imaging and laboratory tests. Medical treatment before and/or after surgery was registered as the duration of different therapies. Mortality was evaluated secondary to Pan-NET disease, and patients with adenocarcinomas or cystic lesions were excluded. The National Population Register was consulted to find out if the included patients were still alive, and the date of death was retrieved if applicable (14). For subsets of cases in which the original pathology report was devoid of relevant information (tumor size, Ki-67 proliferation index), a histopathological re-evaluation was assessed by one of the authors (CCJ).

The Regional Ethical Review Board in Stockholm approved the study, and due to its retrospective nature, no informed consent was required. However, signed informed consent were obtained from the patients prior to surgery.

## Statistics

All proportions were calculated, discounting missing values. Median and interquartile range (IQR) were used. Survival was analyzed with the Kaplan-Meier model, and comparisons were made with the log-rank test. Patients who died without local recurrence or related to Pan-NET were censored to the date of death, and patients were censored to the last follow-up if local recurrence or death had not occurred. Further survival analysis was made with Cox proportional hazard regression. The covariates tumor size, Ki-67 index, and tumor functionality were reported as hazard ratios (HR) with 95% confidence intervals (95% CI). An unpaired two-tailed t-test was also used. Analyses were made using R version 3.6.3 (GUI 1.70 El Capitan build 7735, developed by R Foundation for Statistical Computing, 2016). A *P* -value <0.05 was considered significant.

## Results

**Figure 1:**
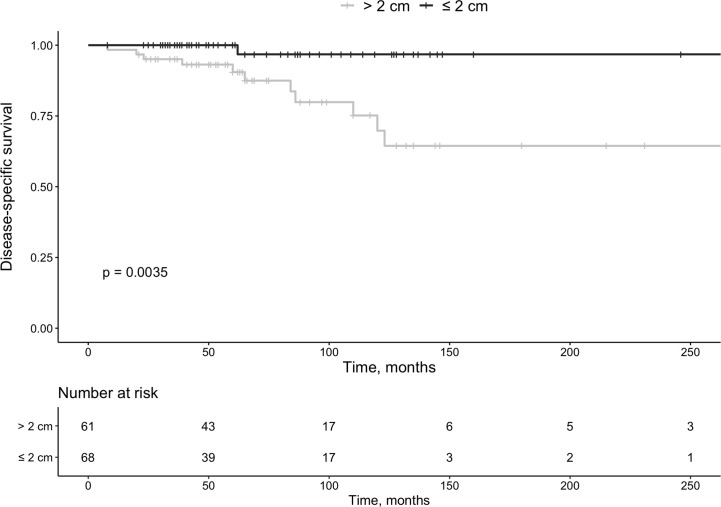
Survival in patients with pan-NETs according to size at diagnosis (<2 cm or >2 cm).

**Figure 2:**
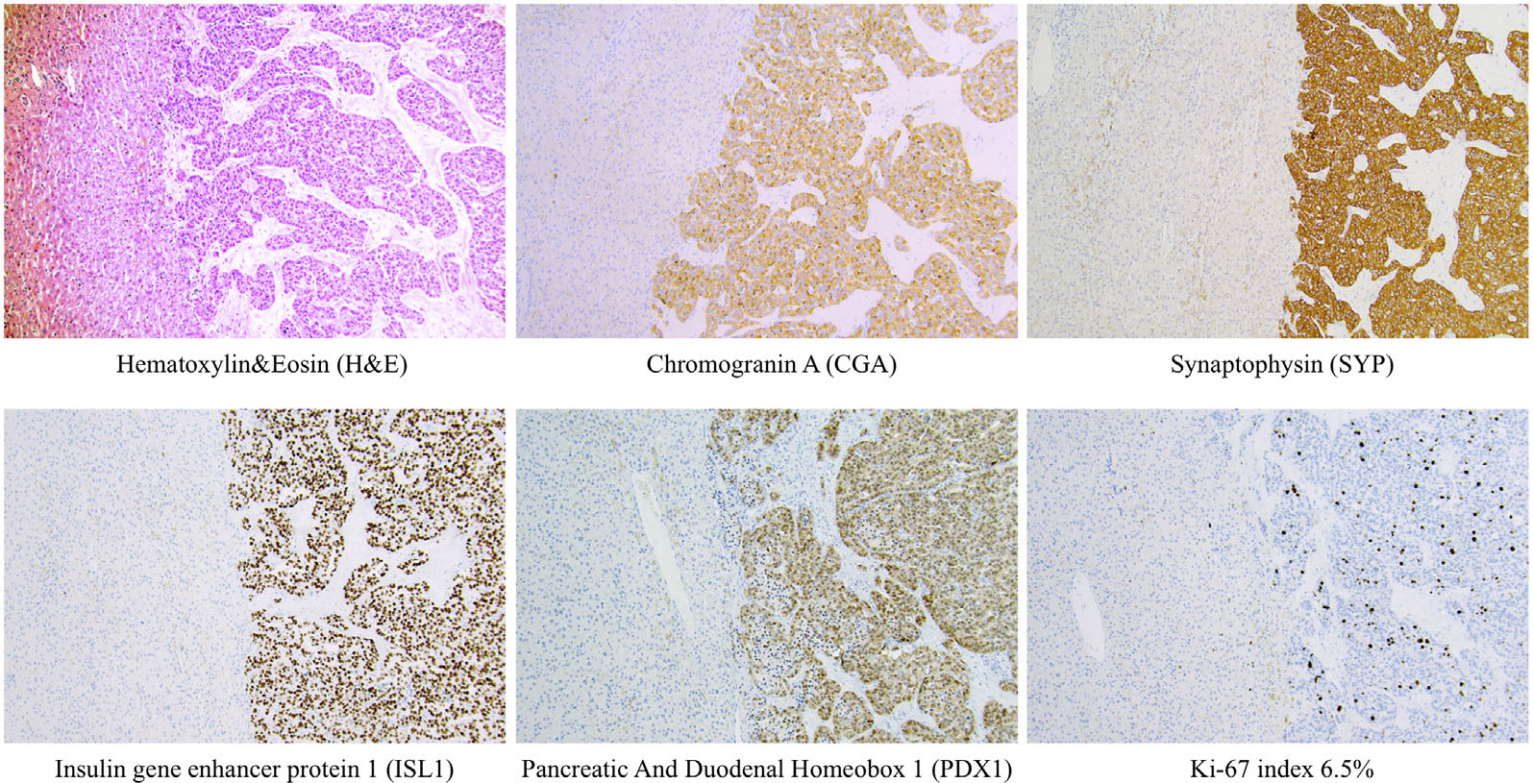
Examples of routine histological and immunohistochemical features of a metastatic pan-NETs WHO grade 2 (Pan-NET G2) from the Karolinska cohort. Metastatic Pan-NETs tissue is evident to the right, while the left section of each image depicts liver tissue. Note the well-differentiated tumoral growth pattern on routine H&E staining. Tumor cells were diffusely positive for markers of neuroendocrine differentiation (CGA, SYP, ISL1) and displayed stainings indicating a pancreatic origin (ISL1, PDX1). The tumor grade was determined to G2, which in this manuscript would translate to the hypothetical G2a category. This patient had been previously diagnosed with a primary pan-NET (data not shown). All photomicrographs were magnified x100.

During the selected time-period, 137 patients (females n=76) were treated and followed for a Pan-NET at the Karolinska University Hospital. Twelve of these patients were diagnosed before 2006. The median age at diagnosis was 61 years (IQR 50 – 71), and tumor size was 2 cm (IQR 1.3-5 cm). Primary tumor size could be evaluated in 129 patients, 61 had tumors ≤ 2 cm, and 68 were > 2 cm (**Table 1**, **2** and **Figure 1**). Tumors were located in the pancreatic tail (cauda) of 63 patients (46%), 41 (30%) in the head (caput), 16 (12%) in the main pancreatic body (corpus) and 10 (7%) had multiple tumors. No detailed anatomic description was recorded in 3 cases (2%), and the remaining tumors were located in the uncinate process. The diagnosis was made using histopathology according to the criteria laid out by the most recent WHO guidelines at that time. An illustration of some key histopathological and immunohistochemical Pan-NET features is provided in **Figure 2**. The Ki-67 index was available from 108 patients and displayed a median value of 2.0% (IQR 1 – 5%) of which 66 cases were G1 and 42 cases G2 tumors. Metastatic disease was present at diagnosis in 22 patients (16%), of which 9 were in G1 tumors, 9 in G2 tumors (*P=*0.051), and the Ki-67 proliferation index was missing in three patients. Fifteen patients (11%) fulfilled the clinical criteria for MEN1 (females n=10), of which three were negative on genetic testing. The median age in patients with MEN1 was 46 years (IQR 37 – 58 years) at diagnosis, which was significantly younger than the rest (*P*<0.001).

**Table 1:** Summary of basal characteristics of 137 patients with pancreatic neuroendocrine tumors at time of diagnosis and follow-up.

	Subjects (%)	Median (IQR)
Females	75 (56%)	
Males	62 (44%)	
Age (years)		61 (50-71)
Size (cm)		2 (1.3-5)
Stage n=137		
Tx, unknown	7 (5%)	
T 1, <2 cm limited to the pancreas	64 ((47%)	
T 2, 2-4 cm limited to the pancreas	9 (7%)	
T 3, > 4 cm limited to the pancreas	35 (26%)	
T 4, invading adjacent organs	22 (16%)	
MEN-1	15 (11%)	
Functioning	30 (22%)	
Surgery	101 (74%)	
Total pancreaectomy	4 (3%)	
Whipple	28 (19%)	
Partial resection	60 (44%)	
Enucleated	6 (4%)	
Liver procedure	3 (2%)	
Re-operated	11 (11%)	
Conservatively (no surgery initially)	36 (26%)	
Surgery at a later stage	2 (4%)	
Follow-up time (months)		74 (41-110)
Deceased	26 (19%)	
Age at death (years)		73 (66-78)
Age at Pan-NET death (years)		75 (66-76)

IQR, interquartile range; MEN-1, multiple endocrine neoplasia; Pan-NET, pancreatic neuroendocrine tumor.

**Table 2:** Initial findings in patients with pancreas neuroendocrine tumors and at follow-up.

	n (%)	Median (IQR)
Patients (n), total	137 (100%)	
G1	66 (48.1%))	
G2	42 (30.6%)	
G2a	30 (21.8%)	
G2b	12 (8.7%)	
Tumor size (cm)		2 (1.3-5)
G1		2.3 (1.5 - 5)
G2		4.3 (1.7-6)
G2a		4 (1.6-6)
G2b		5.5 (3.9-7.3)
Age (years)		61 (50-71)
G1		62 (54-72)
G2		67 (47-69)
G2a		56.5 (48-65)
G2b		61 (46-66)
Ki-67 index at first diagnosis	108 (78.8%)	2 (1-5)
G1		1 (1.1.9)
G2		5.1 (3.5-9.9)
G2a		5 (3.3-6.3)
G2b		11.3 (10-13.5)
Ki-67 index at re-evaluation	26 (18.9%)	9.4 (4-13.8)
G1		4 (3-5.5)
G2		12.5 (9-15.5)
G2a		11.4 (7.8-21.8)
G2b		13 (12.8-14)
Follow-up (months)		74 (41-110)
G1		65 (41-120)
G2		69 (50-92)
G2a		50 (46-96)
G2b		62 (48-83)
Functional tumors (n)	30 (21.8%)	
G1	15 (10.9%)	
G2	10 (7.3%)	
G2a	7 (5%)	
G2b	3 (2.2%)	
Metastasis at diagnosis, total	32 (23.3%)	
G1	9 (6.6%)	
G2	9 (6.6%)	
G2a	6 (4.4%)	
G2b	4 (2.9%)	
Progressive, total*	42 (30.6%)	
G1	19 (13.8%)	
G2	23 (16.7%)	
G2a	14 (102%)	
G2b	9 (6.5%)	

Ki-67 index was evaluated in 108 patients at diagnosis and in 26 during follow-up. All continuous variables are shown as median and interquartile ranges. G1 had a Ki-67 index of <3%, G2 3-20%, G2a 3-9.9% and G2b 10-19.9%. Initial Ki-67 missing in six patients who progressed and by then this index was 10%, 13% and 25%, respectively in three patients. Ki-67 was missing in 5 patients with a functional tumor. F, females. *Patients without initial metastasis. In these patients metastasis developed in median 42 months after surgery (IQR 36-122 months).

Initial surgery was performed in 101 patients 74%; partial pancreatic resection n=60, pancreaticoduodenectomy [Whipple´s procedure] n=26, total pancreatectomy n=4, enucleation n=6, and metastatic liver procedure n= 3. The most commonly used medical therapy was somatostatin analogs (SSA), used in 31 patients (**Table 3**).

**Table 3:** Pharmacotherapy, ablation, embolization and receptor-targeted therapy in pan-NETs patients.

	Total	Treatment before surgery	Treatment inititated after surgery (median months, IQR)	Duration months (IQR)	Treament initiated in non-operated patients (median months, IQR)	Duration in non-operated patients, (median months IQR)
Somatostatin analogs (n)	31	3 (3 - 8 months)	18 (49, 2-105)	66 (21-98)	12 (43, 21-106)	43 (21–106)
Streptozotocin-5-Fluorouracil (n)	9	1	7 (24, 18-102)	9 (6-14)	1	4
Radiofrequency ablation (RF) (n)	7	3 per-operativ	3 (36, 27–57)		1	
Temozolamide (n)	5		4		1	6
Peptide receptor-targeted radiotherapy (PRRT) (n)	5		2		3	
Cisplatin-Etoposide (n)	2		1		1	6
Everolimus (n)	3		1		1	3
Interferon (n)	3		3			
Transarterial embolization, radioembolization (n)	2		2			

Thirty-one were treated with somatostatin analogs, three of these patients were also treated with interferon, three with everolimus, two with temozolomide, and five with peptide receptor-targeted radiotherapy (PRRT). Other treatments according to the table, see text for details.

Preoperatively 3 patients were treated with SSA 3-8 months prior to surgery, 4 were treated with radiofrequency ablation (RF) and 1 with streptozocin-5 FU. After surgery combination therapy was also frequent as three of the 31 somatostatin treated patients were also treated with interferon, three with everolimus, and two with peptide receptor-targeted radiotherapy (PRRT). The most common cytotoxic agents were streptozotocin in combination with 5-fluorouracil in seven patients, which were further combined during follow-up with RF and SSA in three, with cisplatin-etoposide in one and with temozolomide in four patients (**Table 3**). Twelve non-operated patients were treated with SSA, three also with PRRT, and one of these also with everolimus and cisplatin. One of these non-operated with SSA therapy also received 14 months temozolamide. One further non-operated patient was given streptozotocin-5 FU 4 months and later temozolamide for 6 months and a second patient was treated with RF.

**Figure 3:**
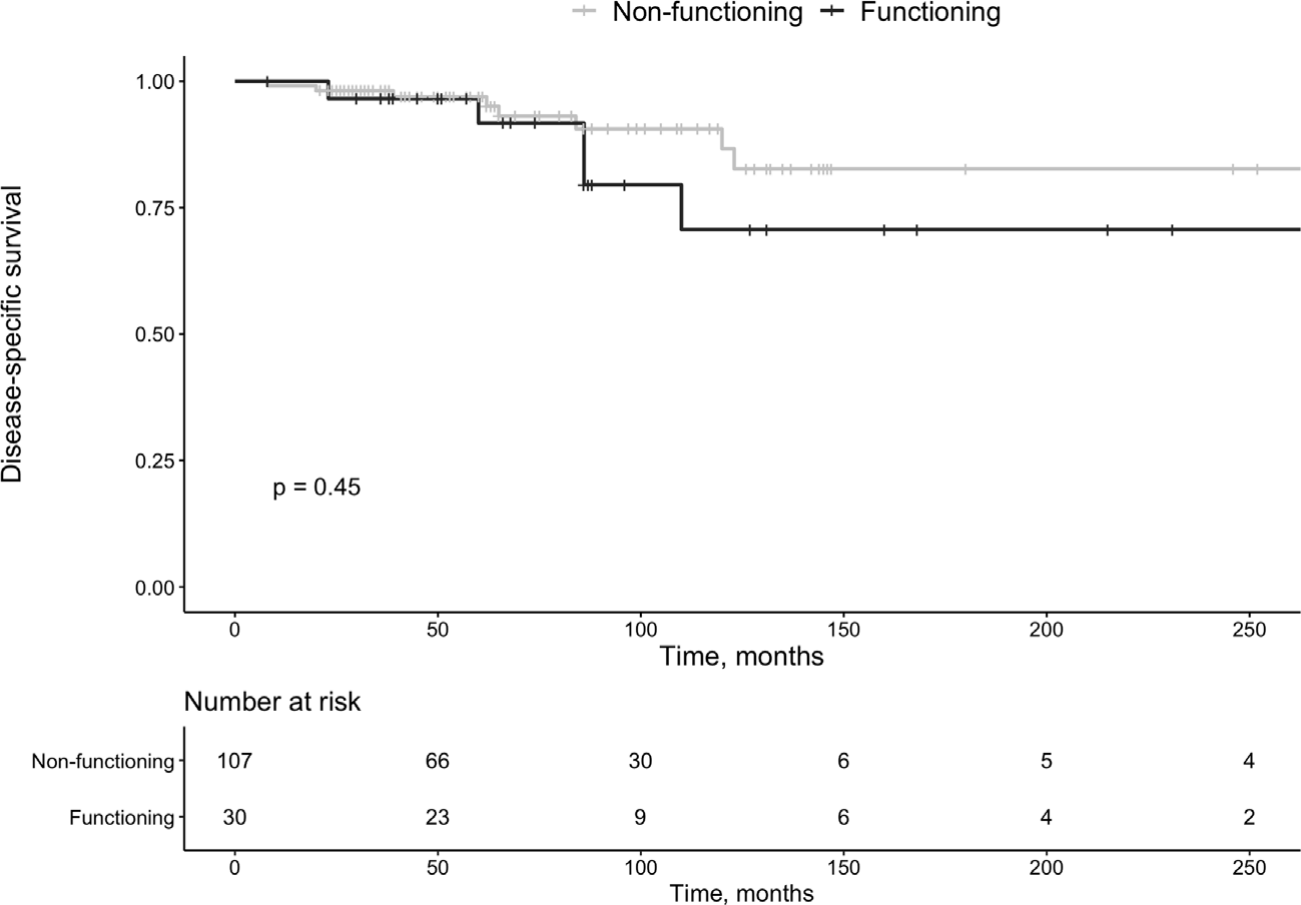
Survival in patients with functioning vs. non-functioning pan-NETs.

Of all tumors, 30 (22%) were hormonally active (insulinomas n=18 [60%], gastrinomas n=4 [13%], somatostatinomas n=2 [7%], VIPoma n=1 [3%], glucagonoma n=1 [3%], PTHrp related hypercalcemia n=1 [3%] and lesions secreting multiple hormones n=3 [10%, gastrin + glucagon n=1, and insulin + glucagon n=2]). Functional tumors were not smaller than non-functional lesions (*P*=0.954), and survival was not associated with a functional tumor (*P*=0.45, **Figure 3**).

### Follow-Up

During the median follow-up of 74 months (IQR 41–110 months), 42 (36%) without previous metastatic disease developed metastases, 19 out of 66 (29%) in G1, and 23 out of 42 (55%) in G2 (*P*= 0.009). All had had primary surgery 45 months (IQR 35-122 months) previously.

**Figure 4:**
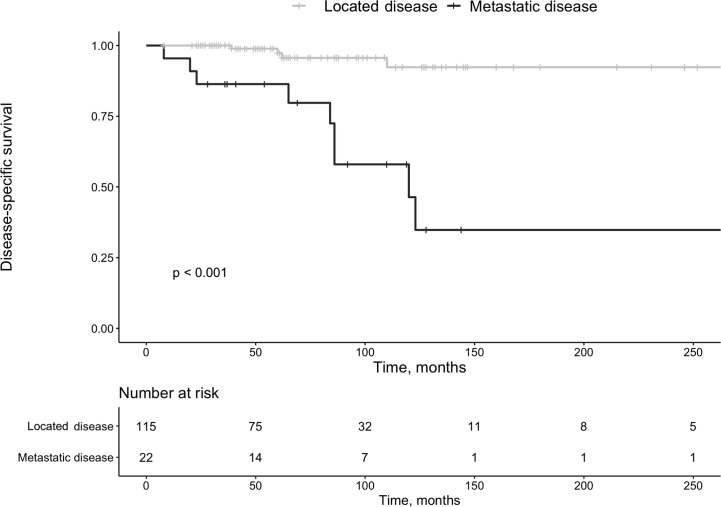
Survival in patients with pan-NETs with localized disease at diagnosis vs. those with metastatic disease at that time point.

Among 22 patients with metastasis at diagnosis, 13 (59%) had further progression during surveillance of 69 months (IQR 37-92 months). Surgery was repeated in 11 of 22 patients 45 months after the initial procedure (IQR 37-92 months). Twenty-six (19%) patients deceased, at a median age of 73 years (IQR 66-78 years) (females, n=16). Ten of 26 deaths (37%) were Pan-NET related (G1 n=2, G2 n=8). Survival was reduced in the patients with metastasis at diagnosis vs. those with localized disease (*P*<0.001, **Figure 4**). Disregarding other mortality causes, Pan-NET specific death in the whole cohort was 7%. Thus, 111 patients (81%) survived during follow-up. Of the Pan-NET associated deaths, 7 out of 10 (70%) cases exhibited malignant insulinomas. Four of these seven (57%) patients with malignant insulinomas had originally non-functioning tumors, which later transformed into insulin-secreting lesions, which have been described in detail elsewhere (15).

Mortality in Pan-NET disease was associated with primary tumor size (*P*=0.035) (**Figure 3**). By Cox-regression using tumor size, Ki-67 index and functionality as covariates, tumor size ≤ 2cm was no longer a significant factor for survival with a HR of 0.13 (95% CI 0.02 – 1.03, *P*=0.053). All patients with MEN1 survived during follow-up (G1 n=7, G2 n=3, and unknown Ki-67 n=5 [small tumors]).

Thirty-six patients of the total cohort (26%) were followed with repeated imaging if they had small non-functioning tumors ≤2 cm (median 1.45 cm, IQR 0.6-1.5 cm). Of these, one had surgery after six years due to radiologic progression from 1.2 to 1.6 cm. Ten patients had no surgery despite a large tumor size (median 5 cm, IQR 3-5.9 cm). Of these, eight had metastatic disease at diagnosis, six were inoperable, one had severe dementia, and one was feeling excellent and did not want any treatment. An 83-year-old patient with an insulinoma hesitant towards surgery was operated two years after diagnosis.

### G1, G2 Tumors and Ki-67

The Ki-67 index was clinically re-analyzed in 26 (19%) patients with progressive disease using core needle biopsy material from metastatic lesions and was at that time median 9.4% (IQR 4 – 13.8%) (**Table 2**). The G1 group included 66 patients (females, n=39) with a median tumor size of 2.25 cm (IQR 1.5-5 cm), and a follow-up of median 48 months (IQR 40.4 – 120 months). Nineteen of these had progressive disease (29%). In the G1 tumors <2 cm distant disease was found during follow-up in 4/32 (13%). The Ki-67 index increased in six tumors which reached a G2 grade and in two reaching a G3 grade. Six patients in the G1 group deceased during follow-up, of which two were Pan-NET associated deaths, and four in causes not related to Pan-NET (breast cancer n=1, glioblastoma n=1, cardiovascular disease n=2).

The G2 group consisted of 42 patients (females, n=22). Patients were in median 57 years old (IQR 47-65 years), median Ki-67 index was 5.1% (IQR 3.5-9.9%), and median tumor size 4.3 cm (IQR 1.7-6 cm). Twelve had metastasis at diagnosis, and 23 (56%) developed progressive disease.

In the further analysis of patients with G2 tumors, the G2a group consisted of 30 patients (females n=16), with a median age of 59.5 years (IQR 51-66.5 years), and follow-up of median 74.5 months (IQR 50 – 106 months). Tumor size was median 4 cm (IQR 1.6-6 cm), and the Ki-67 index was median 4.5% (IQR 3-6.8%). In this cohort, 14/30 (47%) developed progressive disease.

In the G2a patients with progressive disease, the new tumor presentation was local in four and 10 (37%) had distant metastasis. The Ki-67 index increased in these G2a patients to 11.4% (IQR 7.8-21.8%), and one patient progressed to G3. Six patients died during follow-up whereof four related to their Pan-NET and two to unrelated causes (glioblastoma n=1, septicemia n=1).

**Figure 5:**
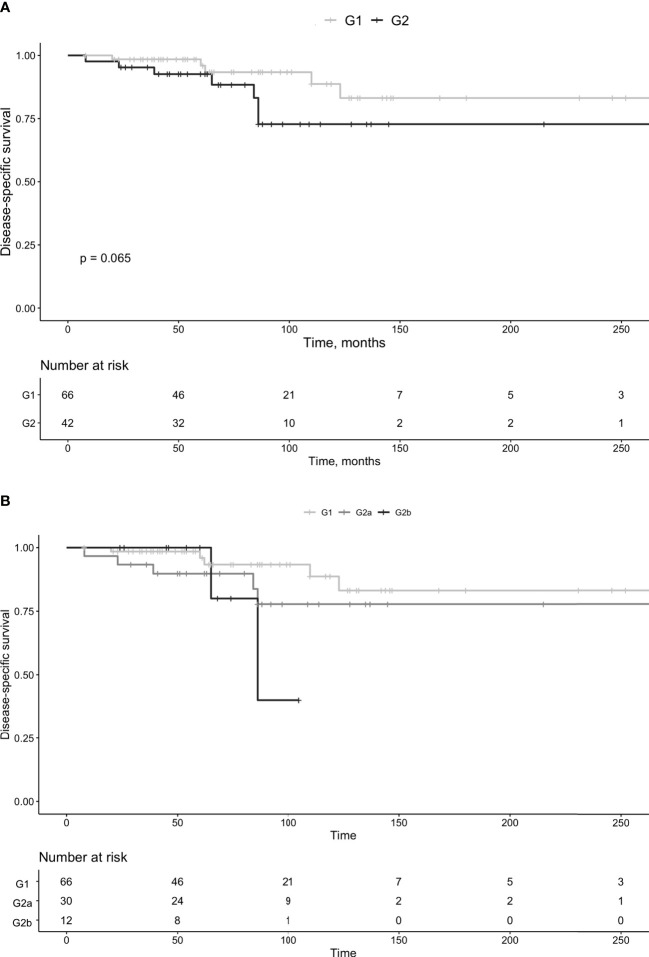
Survival in patients with pan-NETs, divided in G1 and G2 tumors **(A)** and G1, G2a and G2b **(B)**.

Group G2b consisted of 12 patients (females n=6). The median age at diagnosis was 59 years (IQR 46-65 years), and the follow-up was 62.5 months (IQR 48-83 months). Tumor size was 5.5 cm (IQR 3.9-7.3 cm) and the Ki-67 index 11.5% (IQR 10-13.5%). In group G2b, 9 (75%) developed progressive disease and the Ki-67 index in these was 13% (IQR 12.8-14%). No patient progressed to G3. Two G2b patients died during follow-up, of which both deaths were secondary to Pan-NET, combined with liver failure in one. Survival was not statistically different between G1 vs. G2 (*P*=0.065, **Figure 5A**) or G2a vs. G2b (*P*=0.6). Survival in G1 vs. G2 is summarized in **Figure 5A** and in G1 vs. G2a vs. G2b in **Figure 5B**.

## Discussion

This retrospective study from a major tertiary referral center, including 137 patients with Pan-NET followed for 74 months, confirms the notion that patients with metastasis at diagnosis had poor survival compared to those with localized disease. Development of metastatic disease was more frequent in G2 than in G1 tumors, but neither Ki-67 index, tumor functionality, or tumor size were alone or together reliable parameters to assess survival.

Boninsegna et al. have previously found that a Ki-67 of more than 5% is a predictor of recurrent disease (9). In a previous study of 24 patients, re-biopsies of tumor relapse displayed increased Ki-67 counts, from median 4% to 11%, of whom four (17%) progressed to G3 lesions (16). Recently, Botling et al. corroborated this when repeated Ki-67 staining was investigated in 45 patients (17), revealing that 55% of patients progressed during a follow-up of 73 months. In our study, we found some support for this as 31% of patients had progressive disease. In those without a previous metastasis of G1 tumors, 29% developed local or distant recurrence versus 55% of patients with G2 tumors. In the cohort with metastasis at initial diagnosis, 59% of patients had progressive disease. However, better survival in all G1 vs. all G2 (i.e., including those with initial metastasis) did not reach statistical significance (*P*=0.065), probably due to the limited sample size and follow-up time. As the span in proliferation in G2 tumors is wide, ranging from 3-20%, we subdivided this group into hypothetical G2a and G2b groups, but could not display any significant difference in survival after this stratification.

Primary surgery was performed in 74% of patients. Of these, 8% later had repeated surgery due to radiologic progression. Of the remaining, most patients had small non-functional tumors and were followed with clinical and imaging assessment. Seven percent declined follow-up and/or had cardiovascular disease or dementia by which no surgery was performed. There is a controversy whether to operate on patients with tumors between 1-2 cm in size (8), as these often have an indolent behavior (18). However, even small tumors <2 cm can prove to be clinically malignant with distant metastases at diagnosis (19).

During surveillance of small Pan-NETs, a systematic review showed that 0-51% had grown in size during up to 45 months of follow-up, of which 14% of patients had surgery (20). In our investigation, 19 out of 66 (29%) of our patients with G1 tumors developed new local or distant metastasis. In those G1 tumors ≤2 cm in size, this was evident in 4/32 (13%), and the tumor size was missing in four. In two of these small tumors the initial Ki-67 index was lacking. This highlights the importance of adding this information together with radiology and hormone evaluation.

As outlined by Hill and colleagues, surgery is associated with improved survival across all disease stages (21). In that retrospective study, the primary tumor size was not mentioned, but the comparison was made between patient groups that were recommended surgical intervention and had subsequent surgery versus those recommended surgery but did not have surgical intervention (21). When performing surgery in the head of the pancreas, there are risks of gastrointestinal hemorrhage, biliary or gastric outlet obstruction, exocrine and endocrine insufficiency, and the development of a pancreatic fistula (22). These risks have to be considered individually, but also in more advanced disease surgery could be considered to relieve compressive symptoms. Thus, management is controversial in localized tumors in deciding which patients should be recommended surgery. In a retrospective investigation of 125 patients conducted by Phan and co-workers, the best prognosis was observed in patients with clinically indolent disease but also in more aggressive tumors if patients had radical surgery (23). This study differed from ours as 52% of their tumors were functional, and 52% were deemed as malignant. Their median tumor size was 1.9 cm (range 0.3 – 9 cm) in functional tumors and 4 cm (range 0.6 – 18 cm) in non-functional tumors. In other investigations comparing surgery vs. a watchful follow-up in tumors ≤2 cm in Pan-NETs, surgery was not associated with less development of metastasis or death (20, 24).

Larger tumors are more often clinically malignant and have somewhat poorer outcomes, but size alone cannot decide the metastatic potential (25). Tumors >4 cm with invasive characteristics and higher stage have negative prognostic influence, as the 5-year survival in TNM stages I, II, III, and IV were 100%, 93%, 65%, and 35%, respectively (26). In two small investigations (n=9 and n=16, respectively) size was not related to prognosis (27, 28). In contrast, larger tumors were correlated to the development of new lesions in two other investigations (n=108 and n=14, respectively) (29, 30). Recommendations from the National Comprehensive Cancer Network (NCCN) are that tumors ≤2 cm could be considered for observation if discovered incidentally (https://www.nccn.org/professionals/physician_gls/default.aspx, accessed on March 1, 2021). The European Neuroendocrine Tumor Society (ENETS) have proposed intensive observation in non-functional Pan-NETs ≤2 cm (31). In our study, such tumors can progress, but it was rare (13% during 74 months of follow-up). Applying cox-regression, size only showed a trend to be statistically significant. Thus, the approach to have careful surveillance in non-functioning localized tumors ≤2 cm in size seems reasonable. In total, survival varied considerably and of deceased patients, 63% died of causes not related to Pan-NET.

As the Ki-67 index did not accurately pinpoint prognosis in these tumors with heterogeneous activity, clinicians regularly follow these patients. Also, functioning tumors, which are often well-differentiated on histological examination, did not differ from non-functional tumors in terms of survival, and size was not a significant tool using cox-regression. This finding is problematic as non-functioning tumors may change behavior or become functioning (15, 32), and awareness of this is essential if new symptoms or hormone secretion develops.

Other tools, besides the Ki-67 index, tumor size or tumor functionality could be applied. To assess prognosis in NETs, somatostatin receptor PET with low maximum standardized uptake value (SUV_max_) adds information of less progression-free survival and overall survival in a recent review (33). In contrast, a high SUV_max_ on ^18^F-FDG-PET correlates with a more advanced grade (34). To improve prognostication protein expression of DAXX/ATRX (35) and tissue-based markers as transcription factors as ATRX and PDX1 assessed by immunohistochemistry (36), hypermethylation (37) or alternative lengthening of telomeres (38, 39), can be of value but warrants further investigations (40).

There are several limitations to this investigation. Even though we included more patients with Pan-NET than most studies, the sample size is still limited, especially in the subgroups. All data on tumor size and Ki-67 index were not available in the 36 non-operated patients, limiting the evaluation. There is likely a selection bias in those 26 patients with repeated measurements of the Ki-67 index. The 74 months of follow-up is quite long; however, these tumors are slow-growing, and metastasis and death may develop much later, which also may explain difficulties in finding significant findings in the subgroups.

## Conclusion

Our data support the notion that an active follow-up seems reasonable for non-functional Pan-NETs ≤2cm. Progression was more common in G2 tumors than in G1 lesions. Patients with metastasis at diagnosis had poor survival compared to those with localized disease at initial presentation. Taken together, variables such as tumor size, the Ki-67 proliferation index, and hormonal activity may add prognostic value, but no benefits were found by hypothetically subdividing the G2 group. Better clinical prognostic markers are thus needed to assess Pan-NETs in terms of outcome and survival.

## Data Availability Statement

The original contributions presented in the study are included in the article/supplementary material. Further inquiries can be directed to the corresponding author.

## Ethics Statement

The Regional Ethical Review Board in Stockholm, Sweden approved the study (2018/1909-31), and informed consent was waived due to its retrospective nature. All methods were performed in accordance with relevant guidelines and regulations. Written informed consent for participation was not required for this study in accordance with the national legislation and the institutional requirements.

## Author Contributions

JC investigated the medical files and wrote the first draft of the manuscript and the tables. CJ investigated the histopathologic slides and added **Figure 2**. FC made the statistical analyses and contributed with remaining figures. RB added information on surgical considerations. HF aided with continued manuscript revision. All authors contributed to the article and approved the submitted version.

## Funding

This project was supported by grants from the Magnus Bergvall Foundation (Grant Numbers 2017-02138, 2018-02566 and 2019-03149).

## Conflict of Interest

The authors declare that the research was conducted in the absence of any commercial or financial relationships that could be construed as a potential conflict of interest.
